# Vector competence of northern European *Culex pipiens* biotypes and hybrids for West Nile virus is differentially affected by temperature

**DOI:** 10.1186/s13071-016-1677-0

**Published:** 2016-07-07

**Authors:** Chantal B. F. Vogels, Jelke J. Fros, Giel P. Göertz, Gorben P. Pijlman, Constantianus J. M. Koenraadt

**Affiliations:** Laboratory of Entomology, Wageningen University, P.O. Box 16, 6700 AA Wageningen, The Netherlands; Laboratory of Virology, Wageningen University, P.O. Box 16, 6700 AA Wageningen, The Netherlands; Nuffield Department of Medicine, Peter Medawar Building for Pathogen Research, University of Oxford, Oxford, OX1 3SY England UK

**Keywords:** Arbovirus, *Culex*, Vector competence, West Nile virus, Infection, Temperature

## Abstract

**Background:**

Outbreaks of West Nile virus (WNV) have not occurred in northern Europe despite nearby circulation of WNV in the southern part of the continent. The main vector for WNV, the mosquito *Culex* (*Cx.*) *pipiens*, consists of two behaviorally distinct biotypes, *pipiens* and *molestus*, which can form hybrids. Although temperature has been shown to influence vector competence of *Cx. pipiens* for WNV and biotypes are differentially susceptible towards infection, the interaction between the two has not been elucidated.

**Methods:**

We determined vector competence of the *Cx. pipiens* biotypes and hybrids, after 14 days of incubation at 18, 23 and 28 °C. Mosquitoes were orally infected by providing an infectious blood meal or by injecting WNV directly in the thorax. Infection and transmission rates were determined by testing the bodies and saliva for WNV presence. In addition, titers of mosquitoes with WNV-positive bodies and saliva samples were determined.

**Results:**

Orally infected biotype *pipiens* and hybrids showed significantly increased transmission rates with higher temperatures, up to 32 and 14 %, respectively. In contrast, the *molestus* biotype had an overall transmission rate of 10 %, which did not increase with temperature. All mosquitoes that were infected via WNV injections had (close to) 100 % infection and transmission rates, suggesting an important role of the mosquito midgut barrier. We found no effect of increasing temperature on viral titers.

**Conclusions:**

Temperature differentially affected vector competence of the *Cx. pipiens* biotypes. This shows the importance of accounting for biotype-by-temperature interactions, which influence the outcomes of vector competence studies. Vector competence studies with *Cx. pipiens* mosquitoes differentiated to the biotype level are essential for proper WNV risk assessments.

**Electronic supplementary material:**

The online version of this article (doi:10.1186/s13071-016-1677-0) contains supplementary material, which is available to authorized users.

## Background

West Nile virus (WNV; family Flaviviridae) is the most widespread arthropod-borne virus (arbovirus) in the world and can be fatal for humans [1]. The enzootic cycle of WNV is maintained among mosquitoes and birds, whereas mammals (including humans) are dead-end hosts. Outbreaks of WNV have not occurred in northern Europe despite the high abundance of competent mosquito vectors [2, 3], susceptible bird hosts [4, 5], and nearby circulation of WNV in southern Europe [6].

The epidemic potential of WNV is best illustrated by the repeated outbreaks in North America [7]. After the initial introduction of WNV in New York in 1999, WNV rapidly spread across the continent, resulting in the largest outbreak of neuro-invasive disease to date [8, 9]. American passeriform bird species are the main hosts for WNV, with the family of the corvids (Corvidae) being highly susceptible [8, 10–12]. Compared to North America, WNV-associated death rates among birds in southern Europe have been relatively low. It has, therefore, been hypothesized that European bird species are less susceptible to WNV compared to their North American counterparts. Recent studies confirmed, however, that three important bird species originating from Europe, the carrion crow, European jackdaw, and house sparrow, are highly susceptible to WNV [4, 5, 13].

*Culex pipiens* mosquitoes have been identified as the most important vector species for WNV in the United States, because of their vector competence and high abundance during summer [14]. The species *Cx. pipiens* consists of two morphologically identical biotypes, named *Cx. pipiens pipiens* (Linnaeus, 1758) and *Cx. pipiens molestus* (Forskål, 1775), which show distinct behavior [15, 16]. Biotype *pipiens* prefers birds as blood hosts whereas biotype *molestus* prefers mammals [15]. As a consequence, biotype *pipiens* plays an important role in the natural transmission cycle of WNV. Hybrids between the two biotypes have an intermediate host preference, which makes them ideal vectors to bridge WNV from birds to mammals [17]. The presence and abundance of hybrids may, therefore, strongly increase the risk of WNV outbreaks in the human population.

Previous studies showed that proper identification and separation of biotypes is essential, because genetic differences between biotypes and populations influence vector competence [18–20]. Furthermore, our previous studies showed that temperature is an important determinant of vector competence of *Cx. pipiens* mosquitoes for WNV [2, 3]. However, vector competence of the *Cx. pipiens* biotypes has not been tested at different temperatures. Transmission of another arbovirus, the chikungunya virus, has been shown to strongly depend on complex interactions between *Aedes albopictus* populations, viral strains and temperature [21]. The aim of this study was, therefore, to determine the effect of the interaction between temperature and the *Cx. pipiens* biotypes on WNV transmission. Vector competence studies with northern European populations of both *Cx. pipiens* biotypes and hybrids at different temperatures are necessary, to assess the risks for northern Europe in the light of climate change [22, 23].

## Methods

### Mosquitoes

*Culex pipiens* egg rafts were collected during June and July 2014 from aboveground water barrels in Best, The Netherlands. One larva from each egg raft was identified to the biotype level with real-time PCR [24]. *Culex pipiens* colony was started by grouping larvae from 162 egg rafts molecularly identified as biotype *pipiens*. F3 offspring were used for oral virus infection studies, and F5 offspring for virus infection via intra-thoracic injections. *Culex pipiens* biotype *molestus* egg rafts and larvae were collected during September 2013 and February 2014 from an underground habitat in Schiphol airport, Amsterdam, The Netherlands. The colony was started from 38 egg rafts that were autogenously laid by the mosquitoes that emerged from the egg rafts and larvae collected at Schiphol. Real-time PCR confirmed the *molestus* biotype. Crosses between male biotype *pipiens* and female biotype *molestus* resulted in F1 hybrid progeny. The reverse cross between biotypes was not able to produce viable offspring.

All larvae and adults were maintained at 23 °C with 16:8 light:dark photocycle and 60 % relative humidity. Egg rafts were transferred to trays (25 × 25 × 8 cm) filled with water and a drop of Liquifry No. 1 (Interpet Ltd., Dorking, UK). Thereafter, they were daily fed with a 1:1:1 mixture of bovine liver powder, ground rabbit food and ground koi food. Pupae were transferred to Bugdorm cages (30 × 30 × 30 cm) and provided with 6 % *ad libitum* glucose solution. Bovine or chicken blood (Kemperkip, Uden, The Netherlands) was provided through a Hemotek® PS5 feeder (Discovery Workshops, Accrington, UK) for egg production. Female mosquitoes were kept together with males for 4 to 19 days before being transferred to the Biological Safety Level (BSL) 3 facility. Variation in age was kept similar for the two biotypes and their hybrids. To increase mosquito blood-feeding rates, the glucose solution was replaced by water four to six days before the infectious blood meal was offered.

### Virus

In all experiments a P2 stock of West Nile virus lineage 2 originating from Greece (2010) was used with a 50 % tissue culture infective dose ranging between 7.96 × 10^7^ TCID_50_/ml and 1.12 × 10^9^ TCID_50_/ml. WNV was grown on *Ae. albopictus* C6/36 cells and titrated on Green monkey kidney Vero E6 cells. C6/36 cells were cultured with Leibovitz L15 medium (Life technologies, Bleiswijk, The Netherlands) supplemented with 10 % Fetal Bovine Serum (FBS; Life technologies). Vero E6 cells were cultured with Hepes-buffered DMEM medium (Life technologies) supplemented with 10 % FBS, penicillin (100 IU/ml; Sigma-Aldrich, Zwijndrecht, The Netherlands) and streptomycin (100 μg/ml; Sigma-Aldrich). The medium was fully supplemented by adding Fungizone (2.5 μg/ml; Life technologies) and gentamycin (50 μg/ml; Life technologies) when viral infection or titers were determined from mosquito homogenates.

### WNV infection

To determine the period until peak transmission rates and viral titers have developed, we determined viral titers up to 35 days post-infection. Biotype *pipiens* mosquitoes were injected with 69 nl of WNV (1.12 × 10^9^ TCID_50_/ml) using the Drummond Nanoject II Auto-Nanoliter Injector (Drummond Scientific Company, Broomall, PA, United States). Capillaries (3.5” Drummond # 3-000-203-G/X, Drummond Scientific Company) were drawn to a fine point with the Narishige needle puller model PB-7 (Narishige, Tokyo, Japan). Injections instead of infectious blood meals were used in order to obtain 100 % infection [2]. Injected mosquitoes were divided over 6 groups of 24 female mosquitoes, and placed in buckets (Ø: 12.5 cm, height: 12.5 cm) at 23 °C for 7, 14, 21, 28 or 35 days. One group of female mosquitoes was directly killed (day 0) and individually stored at -80 °C.

The *Cx. pipiens* biotypes and hybrids were orally infected with WNV in order to test for the effect of temperature on infection and transmission rates [2]. In the BSL3 facility, biotype *pipiens*, biotype *molestus*, and hybrid mosquitoes were kept in groups of 20–100 females in buckets. Female mosquitoes were allowed to feed for one hour on infectious chicken blood with a titer of 5.7 ± 1.0 × 10^7^ TCID_50_/ml, offered through a Hemotek PS5 feeder. The blood meal was offered during the early stage of the dark period in a dark room at 24 °C and 60 % RH. After one hour, mosquitoes were immobilized on a semi-permeable CO_2_-pad connected to 100 % CO_2_. Blood-fed mosquitoes of each group were selected and divided in smaller groups of maximum 20 female mosquitoes over three separate buckets. The buckets were placed at 18, 23 or 28 °C for 14 days. Oral infections were replicated four to seven times until a total number of 50 female mosquitoes of each biotype per temperature was reached.

To determine the effect of the midgut on WNV dissemination, mosquitoes were also injected with WNV. Biotype *pipiens*, biotype *molestus*, and hybrid mosquitoes were immobilized with CO_2_, and injected in the thorax with 69 nl of WNV (1.12 × 10^9^ TCID_50_/ml). Mosquitoes from each group were divided in groups of maximally 12 female mosquitoes over three separate buckets and placed at 18, 23 or 28 °C for 14 days. Injections were replicated three times until a total number of 20 ± 1 female mosquitoes of each biotype per temperature was reached.

### Infection and transmission rates

After the incubation period of 14 days, mosquitoes were immobilized with CO_2_ and legs and wings were removed. To collect saliva, the proboscis of each mosquito was inserted in a pipette tip with 5 μl of a 1:1 solution of FBS and 50 % glucose solution, for a minimum period of 45 min. Each saliva sample was collected in a 1.5 ml Eppendorf safe-lock microcentrifuge tube with 55 μl of fully supplemented Hepes-buffered DMEM medium, whereas bodies were individually stored in 1.5 ml Eppendorf safe-lock microcentrifuge tubes with a small scoop of 0.5 mm Zirconium beads (Bio-Connect BV, Huissen, The Netherlands). All samples were stored at -80 °C until further use.

Frozen mosquito bodies were homogenized in the Bullet blender storm (Next Advance, New York, United States) for 2 min, and shortly spun down at 14,000 rpm. Next, 100 μl of fully supplemented Hepes-buffered DMEM medium was added and samples were again homogenized for 2 min in the Bullet blender, and spun down for 2 min at 14,000 rpm. For each mosquito homogenate or saliva sample, 30 μl was incubated on a monolayer of Vero E6 cells in a 96-well plate. After 2–3 h the medium was completely removed and replaced by 100 μl of new medium. After three days, each well was scored for cytopathic effects specific for WNV. Titers were determined by endpoint dilution assays on Vero E6 cells, and scored for cytopathic effects after 3 days.

### Statistical analysis

Generalized linear models with a binomial distribution and logit link function were used to test for the effects of biotype and temperature on the WNV infection and transmission rates, for the orally infected mosquitoes. Mosquitoes with positive saliva, but negative body (1 %) were excluded from the analysis. Infection and transmission rates were calculated, respectively, by dividing the number of female mosquitoes with infected bodies or with infected saliva by the total number of female mosquitoes in the respective treatment. Effects of replicate, biotype, temperature, and the interaction term between biotype and temperature, were included in both models. When interaction terms were significant, the effect of temperature was tested separately for each biotype with likelihood ratio (LR) tests. Multiple comparisons were corrected with the Bonferroni procedure, by lowering the significance level from 0.05 to 0.017. The effect of temperature on viral titers was tested separately for each biotype with Wilcoxon or Kruskal-Wallis tests. All statistical analyses were done with SAS software, version 9.3 (SAS Institute Inc., Cary, USA), and figures were created with the statistical software package R (R foundation for Statistical Computing, Vienna, Austria).

## Results

**Fig. 1:**
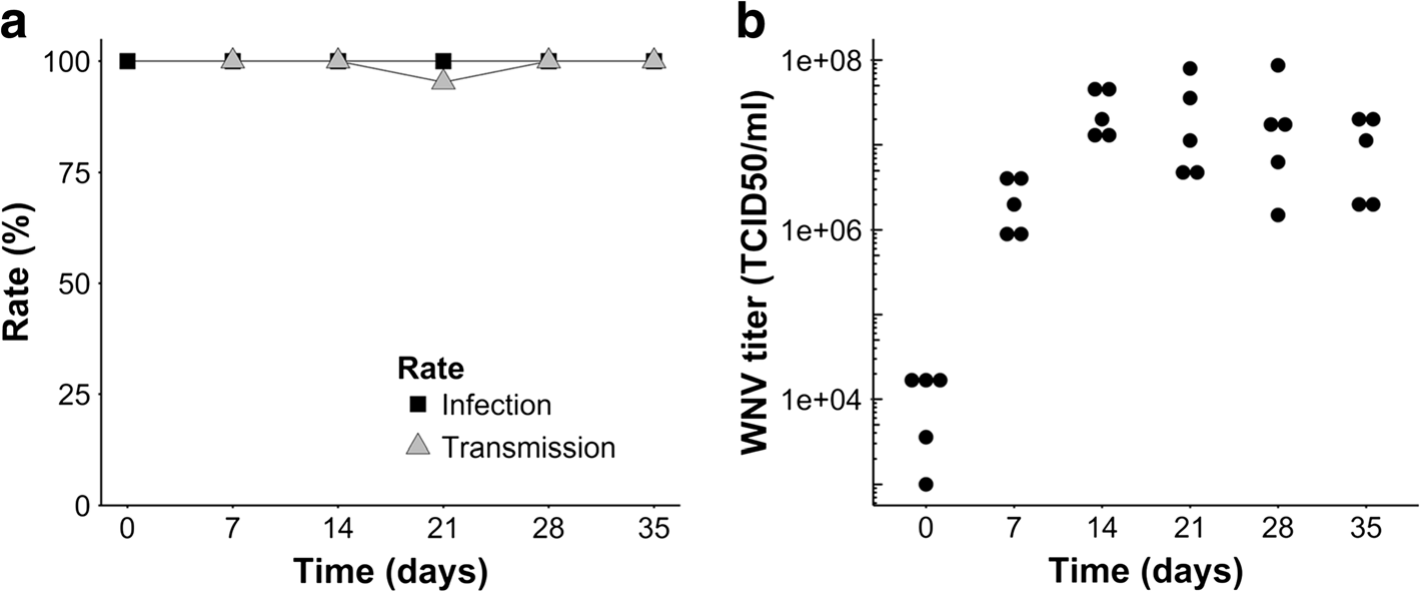
Vector competence and titers of WNV injected *Cx. pipiens* biotype *pipiens* females over 35 days. Infection (*squares*) and transmission (*triangles*) rates of biotype *pipiens* females incubated at 23 °C from day 0 to 35 post-injection (**a**). Each data point represents 16–24 mosquitoes. WNV titers of five selected female mosquitoes at each time point from day 0 to 35 post-infection (**b**)

To determine the minimum incubation period until peak viral titers were reached, viral titers were assessed for *Cx. pipiens* biotype *pipiens* females up to 35 days post-WNV infection (Additional file 1: Table S1). Infection rates were 100 % at all time points from day zero to 35 post-infection (Fig. 1a). In addition, at all time points all mosquitoes tested had virus-positive saliva except for one female at day 21 (Fig. 1a). After 14 days, viral titers of the mosquito bodies were approximately 1,000-fold higher than titers at the day of infection, and remained high over time (average titer of 2 × 10^7^ TCID_50_/ml; Fig. 1b).

**Fig. 2:**
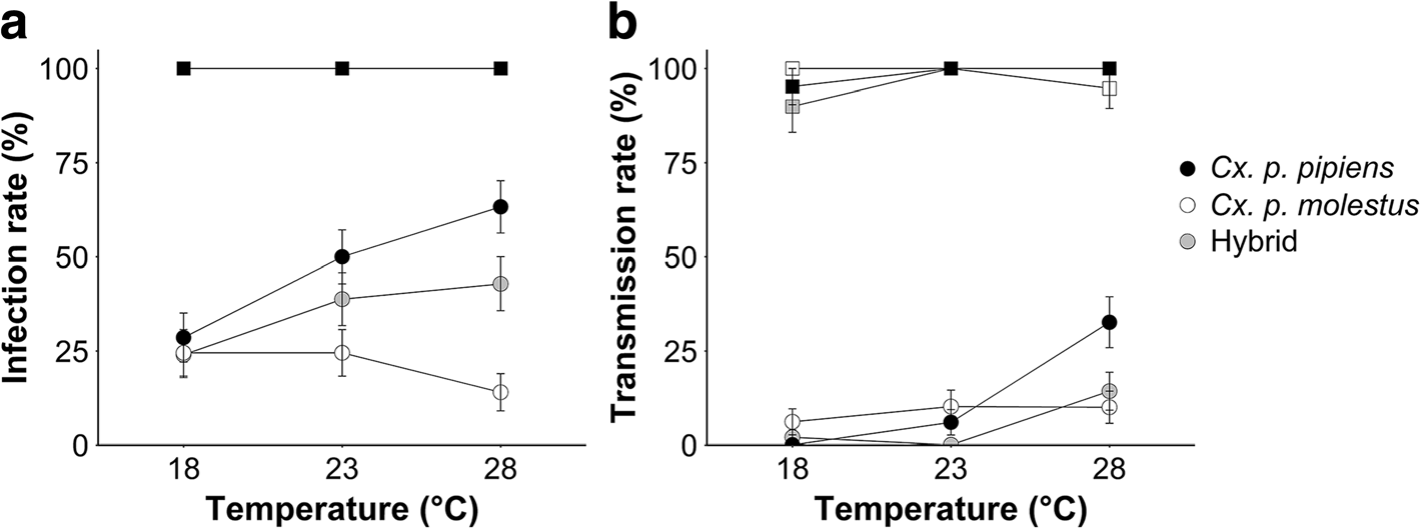
Vector competence of the *Cx. pipiens* biotypes and hybrids incubated at 18, 23, or 28 °C for 14 days. Infection rates (**a**) and transmission rates (**b**) of biotype *pipiens* (*black*), biotype *molestus* (*white*), and hybrids (*grey*) after oral infection (*circles*) and injections (*squares*). Data points of the biotypes and hybrids after injections are overlapping. Each data point represents 50 female mosquitoes for oral infections, and 19–21 female mosquitoes for injections. Error bars show standard error of the means

Infection and transmission rates were determined for orally infected biotype *pipiens,* biotype *molestus*, and hybrid females after 14 days of incubation at 18, 23 or 28 °C (Additional file 1: Table S2). Initial model analyses of infection rates showed a significant interaction effect between biotype and temperature (GLM, *χ*^2^ = 12.89, *df* = 4, *P* = 0.012), which indicated that infection rates of biotypes responded differently to temperature (Fig. 2a). Selections were, therefore, made to test for the effect of temperature separately for each biotype. Infection rates were significantly different between temperatures for biotype *pipiens* (28.6–63.3 %; Likelihood ratio test (LR), *χ*^2^ = 12.54, *df* = 2, *P* = 0.002), but there was no significant difference for biotype *molestus* (24.5–14.0 %; LR, *χ*^2^ = 3.96, *df* = 2, *P* = 0.14), and hybrids (24.0–42.9 %; LR, *χ*^2^ = 5.07, *df* = 2, *P* = 0.080). For biotype *pipiens*, the infection rate was significantly higher at 28 °C compared to 18 °C (LR, *χ*^2^ = 12.31, *df* = 1, *P* < 0.001), but not at 23 °C compared to 18 °C (LR, *χ*^2^ = 4.91, *df* = 1, *P* = 0.027; threshold *P*-value = 0.017 after Bonferroni correction), and 23 compared to 28 °C (LR, *χ*^2^ = 1.79, *df* = 1, *P* = 0.18). Thus, infection rates of biotype *pipiens* increased with higher temperatures, with the strongest increase at 28 °C, whereas there was no effect of temperature on infection rates of biotype *molestus* and hybrids.

To determine whether a female mosquito can actually transmit WNV, transmission rates were determined based on the presence of WNV in the saliva. Model analyses again showed a significant interaction effect between biotype and temperature (GLM, *χ*^2^ = 15.71, *df* = 4, *P* = 0.003), which indicated that also transmission rates of the biotypes responded differentially to temperature (Fig. 2b). Therefore, the effect of temperature on the transmission rate of each biotype was tested separately. Transmission rates significantly increased with temperature for biotype *pipiens* (0–32.7 %; LR, *χ*^2^ = 29.95, *df* = 2, *P* < 0.001) and hybrids (0–14.3 %; LR, *χ*^2^ = 13.49, *df* = 2, *P* = 0.001), but not for biotype *molestus* (6.1–10.2 %; LR, *χ*^2^ = 0.77, *df* = 2, *P* = 0.68). For biotype *pipiens*, the transmission rate was significantly higher at 28 °C compared to 18 °C (LR, *χ*^2^ = 26.41, *df* = 1, *P* < 0.001) and compared to 23 °C (LR, *χ*^2^ = 12.75, *df* = 1, *P* < 0.001), but not at 23 °C compared to 18 °C (LR, *χ*^2^ = 4.29, *df* = 1, *P* = 0.038; threshold *P*-value = 0.017 after Bonferroni correction). Also for hybrids, the transmission rate was significantly higher at 28 °C compared to 18 °C (LR, *χ*^2^ = 6.58, *df* = 1, *P* = 0.010) and compared to 23 °C (LR, *χ*^2^ = 11.18, *df* = 1, *P* < 0.001), but there was no difference between rates at 18 and 23 °C (LR, *χ*^2^ = 1.35, *df* = 1, *P* = 0.24). Thus, transmission rates of biotypes responded differently to temperature, with biotype *pipiens* and hybrids showing a significant increase with higher temperature, whereas there was no effect of temperature on biotype *molestus.*

In order to test whether different responses of biotypes to temperature could be attributed to the midgut barrier or the salivary gland barrier, the *Cx. pipiens* biotypes and hybrids were injected with WNV. By injecting WNV directly in the thorax the midgut barrier is bypassed and only the salivary gland barrier needs to be overcome before WNV can be transmitted. After 14 days of incubation, the infection rates were 100 % for both biotypes and hybrids, at all temperatures (Fig. 2a). Transmission rates of female mosquitoes at most temperatures were (close to) 100 % (Fig. 2b), suggesting that there is no apparent salivary gland barrier.

**Fig. 3:**
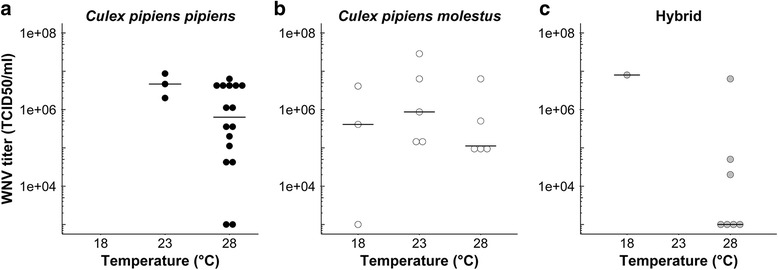
WNV titers of orally infected *Cx. pipiens* females with virus-positive bodies and saliva. WNV titers were determined after 14 days of incubation at 18, 23, or 28 °C for biotype *pipiens* (**a**), biotype *molestus* (**b**) and hybrids (**c**). Horizontal black lines show median titers at each temperature

Finally, viral titers of all orally infected female mosquitoes with virus-positive body and saliva were determined. The effect of temperature on viral titer was again tested separately for each biotype. There was no significant effect of temperature on median viral titers of biotype *pipiens* (Wilcoxon test, *Z* = 1.51, *df* = 1, *P* = 0.13; Fig. 3a), biotype *molestus* (Kruskal-Wallis test, *χ*^2^ = 1.96, *df* = 2, *P* = 0.38; Fig. 3b), and hybrids (Wilcoxon test, *Z* = 1.40, *df* = 1, *P* = 0.13; Fig. 3c). These results demonstrate that viral titers did not increase with higher temperatures, indicating that after successfully passing the midgut barrier there is no difference between the biotypes.

## Discussion

The aim of this study was to investigate the interaction between temperature and *Cx. pipiens* biotypes on WNV transmission. We found that temperature differentially affected vector competence of the *Cx. pipiens* biotypes and their hybrids. Transmission rates of biotype *pipiens* (0–32 %) and hybrids (0–14 %) increased with higher temperature, especially at 28 °C, whereas transmission rates of biotype *molestus* (6–10 %) were not affected by temperature. Transmission rates of biotype *pipiens* were within the range of other *Cx. pipiens* populations in WNV endemic areas [25–27]. Peak transmission rates of biotype *molestus* and hybrids were, however, two- to three-fold lower.

Positive relations between temperature and vector competence are not limited to WNV and *Cx. pipiens* [2, 27], but have also been described for other virus-vector systems [3, 21]. This positive relation is most likely the result of increased replication of the virus at higher temperatures [3, 28, 29]. Interestingly, the transmission rates of biotype *molestus* did not increase with higher temperature. In this study, all female mosquitoes were reared under the same controlled conditions. Observed differences between biotypes are, therefore, likely due to interactions between genotype and temperature. When bypassing the midgut barrier by injecting WNV in female mosquitoes, transmission rates were similar for the biotypes and their hybrids. This suggests that genetic factors determining midgut characteristics can explain why temperature had no effect on transmission rates of biotype *molestus*. The highest temperature may have induced changes in the morphology and physiology of the midgut of biotype *pipiens* and hybrids. As a result, the effectiveness of the midgut as a barrier may have decreased, whereas there seemed to be a higher threshold for biotype *molestus*. Such lowered effectiveness of the midgut barrier at higher temperatures has been shown with *Ae. albopictus* mosquitoes infected with dengue virus [30]. Alternatively, temperature may have induced changes in the regulation of biotype-specific immunoresponsive genes, as has been shown for *Aedes aegypti* mosquitoes infected with Chikungunya virus [31]. In addition, the RNAi pathway becomes more active at higher temperatures and can inhibit viral replication [32]. Differences in RNAi activity between the biotypes may have resulted in increased sensitivity for WNV infection of biotype *pipiens* and hybrids at higher temperatures, whereas such changes were not induced in biotype *molestus*. Future studies should focus on the underlying mechanisms in the midgut that can explain why vector competence of biotype *molestus* for WNV did not increase with higher temperature.

Vector competence of *Cx. pipiens* for WNV varies in time and among different mosquito populations [33]. Vector competence of North American lines of biotype *pipiens* (Pennsylvania) and of biotype *molestus* (New Jersey) populations were equal, but lower than vector competence of their hybrid progeny [19]. In contrast, significant differences in vector competence were found between biotype *pipiens* (Woodland) and biotype *molestus* (Sacramento) populations [20]. No differences in transmission rates were found between four different autogenous and anautogenous *Cx. pipiens* populations in Italy [26]. Our study shows that such variation can be partly explained by temperature, but other complex interactions between viral strains, viral titers, and geographically distinct vector populations have been shown to play a role as well [2, 21, 34]. In addition, colonization in the laboratory could have influenced the outcomes of these vector competence studies [35–38]. Such effects have been shown to occur after eight generations for *Cx. pipiens* mosquitoes infected with Rift Valley Fever virus [35], but this is dependent on the number of females the colony was established with, and selection pressures in the rearing environment [36, 37]. In order to fully understand factors that are required for WNV transmission, a direct comparison should be made between vector competence of recently established mosquito populations originating from endemic and non-endemic areas at different temperatures.

Vector competence is only one qualitative component of the more complex, quantitative vectorial capacity of a species to transmit pathogens [39, 40]. Vectorial capacity also takes into account other factors such as vector and host abundance, host preference, biting rate and survival. Our results confirm that temperature is an important factor that may explain the current absence of WNV transmission in northern Europe. Temperature is, however, not the only factor that can predict when and where WNV transmission cycles can be established [41]. In order to determine the risk of WNV establishment and further transmission, it is essential to use valid predictors of all components of vectorial capacity in risk models. Our results emphasize the need to characterize *Cx. pipiens* populations to the biotype level for reliable assessments of the risk for future WNV outbreaks.

Based on our findings that there is an increased likelihood of WNV transmission by *Cx. pipiens* at higher temperatures, and climate change predictions of longer and more intense heat waves during summer [22, 23], future WNV transmission in northern Europe cannot be ruled out. Preparing for future WNV transmission in northern Europe is, therefore, advised.

## Conclusion

Vector competence of the *Cx. pipiens* biotypes and their hybrids for WNV is differentially affected by temperature. Transmission rates of biotype *pipiens* and hybrids increased with higher temperatures, but not so for biotype *molestus*. Our study emphasizes the need to identify *Cx. pipiens* mosquitoes to the biotype level because of the differential vector competence and vectorial capacity for WNV.

## Abbreviations

Arbovirus, arthropod-borne virus; BSL, biological safety level; FBS, fetal bovine serum; GLM, generalized linear model; LR, likelihood ratio test; TCID_50_, 50 % tissue culture infective dose; WNV, West Nile virus

## Additional file

Additional file 1: Table S1. Supporting data for experiment on incubation period until peak WNV titers. **Table S2.** Supporting data for experiment on vector competence of *Cx. pipiens* biotypes for WNV. (XLSX 73 kb)
